# The relation between cigarette taxes and older adult smoking in Zhejiang and Gansu: what happened following the 2009 Chinese Tax adjustments?

**DOI:** 10.1186/s12199-017-0640-9

**Published:** 2017-04-04

**Authors:** Qing Wang

**Affiliations:** School of business, Dalian University of Technology, Panjin, 124221 Liaoning China

**Keywords:** Cigarette excise tax rates, Smoking, Daily consumed cigarettes number, Self-reported health, Chronic disease, China

## Abstract

**Background:**

In May 2009, the Chinese government raised cigarette excise tax rates and adjusted standards for Grade A cigarettes and Grade B cigarettes. The present study aimed to examine the effects of the tax adjustments in 2009 on smoking behaviors and health outcomes among smokers aged above 45.

**Methods:**

Data from the 2008 and 2012 China Health and Retirement Longitudinal Study of Zhejiang and Gansu provinces were used to estimate the influence of tax increase on the number of cigarettes smoked daily and health capital. The sample included 706 smokers who were 45 years old and older at the time of data collection in 2008. The sample group was surveyed again in 2012. The final sample size was 1366. Logit model was applied.

**Results:**

Cigarette tax adjustment in 2009 resulted in the decrease in the likelihood of smoking 0–10 cigarettes per day by 1.06%; the increase in the likelihood of those smoking 11–20 cigarettes per day by 0.44%; and, those smoking 20 cigarettes or more by 0.63%; the decrease in the likelihood of good health by 0.47%; the increase in the prevalence of chronic disease by 1.34%.

**Conclusions:**

The smoke tax adjustment in 2009 worsened individual unhealthy smoking behaviors and health outcomes. The proposed cigarette tax levied at the retail level can reduce the State Tobacco Monopoly Administration’s control overall and each price and increase the influence of the market on cigarette consumption in China.

## Background

China is the largest country in terms of both cigarette consumption and production. So far, China has 350 million smokers, making up approximately one third of smokers around the world. Furthermore, about 30% of all cigarettes on the earth are produced in China [1]. Smoking has resulted in tremendous health risks in China with annual 1.36 million deaths [2]. Recognizing these risks, China signed the World Health Organization (WHO) Framework Convention on Tobacco Control (FCTC) on November 11, 2003, and it went into effect on January 9, 2006. Since then, the Chinese government has adopted multiple strategies for tobacco control.

Taxation is generally regarded as one of the most effective interventions to control tobacco use [3]. In comparaison of other smoking control measures such as warnings or education, taxation work as a direct way to curb tobacco demand by price inflation of tobacco products. It has been well-established that an increase in tax reduces tobacco consumption, decreases the prevalence of tobacco use, decreases smoking initiation, and increases the likelihood of smoking cessation [4–13]. However, recent evidence casts some doubt into these conclusions. Some of the studies claimed no effect on smoking initiation and quit ratio [13–17].

For the moment, tobacco tax rates in China lag far behind the world average, leading to wide and easy access to tobacco products [18]. The actual level is approximately 46% even after the adjustment of the tobacco excise tax in 2009, while according to the data from WHO, the average level worldwide has reached 67%, and the rate in 33 countries has over 75% [19]. Therefore, a significant potential exists to prompt tobacco control in China by increasing taxes on tobacco products, which is also encouraged by WHO. Given the cigarette monopoly system, China’s tobacco pricing mechanism is unique. However, no studies have reported the effectiveness of the tax adjustments in China. The present study aimed to estimate the impacts of the tax adjustments in 2009 on smoking behaviors and health outcome.

### China’s cigarette pricing mechanism

Most studies motioned above have been carried out among high-income countries with market economies instead in contexts such as China adopting a system of unified leadership, vertical management and monopolized operation. Chinese National Tobacco Company (CNTC), a government-owned organization, monopolized all cigarette production and wholesalers markets in China. State Tobacco Monopoly Administration (STMA) has oversight over CNTC. STMA regulates the tobacco monopoly by making cigarette “allocation plans” as well as the wholesale-retail profit margin and allocation-wholesale profit margin. Cigarette producers provide cigarettes to wholesalers at the allocation price including the excise tax. The retail price equaled the allocation price plus the allocation-wholesale margin, the wholesale-retail margin, and the value-added tax (VAT = 17%). Given the retail price, the wholesale price and allocation price are fixed accordingly. Similarly, once the allocation price is determined, the STMA can easily control wholesale and retail price by adjusting the allocation-wholesale margin and wholesale-retail margin as it desires [18].

### The 2009 Chinese Tax adjustments on cigarettes

Cigarettes are graded into Grade A and Grade B for the convenient of tax collection using allocation price as the grading standard. Different excise tax and ad valorem tax rates are applied to Grade A and Grade B cigarettes. Before 2009, cigarettes with allocation prices of 5 yuan and above per pack (20 cigarettes) were classified as Grade A with 45% excise rate at producer level, and those with allocation prices below 5 yuan were categorized as Grade B with 30% excise tax rates. In May 2009, the Chinese government issued and implemented Taxation Legislation No. 84, The Notice Regarding Adjustment to Tobacco Product Excise Tax Policy. The following lists the tax adjustment items in 2009: (1) an raise in excise tax rates from 45 to 56% for Grade A cigarettes and from 30 to 36% for Grade B cigarettes; (2) standards for Grade A cigarettes and Grade B cigarettes were rearranged such that cigarettes with allocation prices of 7 yuan and above per pack (20 cigarettes) were classified Grade A, and those with allocation prices below 7 yuan were regarded as Grade B. Therefore, the excise tax rates were increased from 45 to 56% for allocation price higher than 7 yuan, and from 30 to 36% for allocation price lower than 5 yuan, while the rate decreased from 45 to 36% for allocation price between 5 and 7 yuan.

At the same time, the STMA issued document No. 180 (2009), Notice of Adjusting Cigarettes Allocation Price. Accordingly, the allocation-wholesale profit margin was decreased for each cigarette class. Table 1 presents cigarette classification and profit margins before and after 2009 (see Table 1).

**Table 1:** Cigarette Classification and Profit Margins

Allocation price per pack	Grade		Excise Tax Rate at producer level		Allocation-wholesale margin		Wholesale-retail margin	
	After 2009	Before 2009	After 2009	Before 2009	After 2009	Before 2009	After 2009	Before 2009
≥10 Yuan	A	A	56%	45%	46%	47%	10%	10%
≥7 Yuan and < 10 Yuan	A	A	56%	45%	33.33%	43%	10%	10%
≥5 Yuan and < 7 Yuan	B	A	36%	45%	33.33%	43%	10%	10%
≥3 Yuan and < 5 Yuan	B	B	36%	30%	33.33%	38%	10%	10%
≥1.65 Yuan and < 3 Yuan	B	B	36%	30%	25%	28%	10%	10%
<1.65 Yuan	B	B	36%	30%	17.65%	18%	10%	10%

The cigarette retail prices increased slightly from 6.26 yuan before the tax adjustments to 6.40 yuan after 2009 [20]. The increased retail price may lead to decreased tobacco consumption. But the tax rate did not increase for all the tobacco class. The tax rate and profit margins decreased for allocation price between than 5 and 7 yuan, therefore, the retail price decreased for such cigarettes, which may lead to compensatory behavior among smokers. In order to maintain the affordability of tobacco products, some smokers may choose to purchase from low-taxed and untaxed sources of cigarette for allocation price between than 5 and 7 yuan. The compensatory of smokers substituting cheaper tobacco products, consuming from low-taxed and untaxed sources of cigarette had been widely found [21–32]. The compensatory behavior may lead the taxation of tobacco products not to be as effective in curbing tobacco consumption as it is intended to be and diminish the expected reduction in cigarette consumption and in turn, dampen the impact of any tax-induced price increase on public health outcomes.

The previous study using retail price as the main mechanic to estimate the influence of tax adjustments on cigarettes, which may cannot take the latter impact into consideration. Consumers may change cigarettes brand to keep price unchanged before and after 2009. This study used tax rate before and after 2009 to evaluate the effect of tax adjustment related to price change and compensatory behavior with retail price controlled. This is the first study to quantify the effects of cigarette tax adjustment in 2009 on smoking behaviors and health of a large representative sample in China.

## Methods

### Data

This study used data from the 2008 and 2012 China Health and Retirement Longitudinal Study (CHARLS) of Zhejiang and Gansu provinces. Gansu province is located in the economic and social undeveloped western area, Zhejiang in east developed region. These two provinces were chosen to get at extremes within China. The CHARLS adopted a multi-stage, stratified probability-proportionate-to-size (PPS) sampling. Counties were chosen by PPS stratified based on urban or rural regions. For details, see http://charls.ccer.edu.cn/zh-CN. The study was approved by the Institutional Review Board of Peking University with ethical approval NO. (IRB00001052-11014).

The sample included those who were 45 years old or older at the time of data collection in 2008. The 2008 sample comprised 706 smokers in 2685 individuals (observation with missing value were excluded). The survey was followed in 2012. Due to dropout, 23 respondents were not observable in 2012. Among 706 smokers in 2008, only 683 smokers’ information was collected in 2012. Observations surveyed in 2008 but no followed in 2012 were excluded, and the final sample size was 1366 smokers.

### Measures

#### Smoking and health outcome measures

Three independent variables were examined. The first was “daily consumed cigarettes number,” based on the answers to CHARLS question “How many cigarettes do you smoke each day now?” “Daily consumed cigarettes number" was classified into an ordinal variable from 1 to 3 representing the level of smoking (1 = smoked cigarettes 0 – 10 per day; 2 = 11 – 20 cigarettes per day, 3 = more than 20 cigarettes per day). The second was self-reported health status. The CHARLS survey asked each respondent the following question: ”How would you evaluate your health? Excellent, very good, good, fair, poor, or very poor?“ “Self-reported health” was recoded into an ordinal variable from 1 to 3 representing the level of health (1 = excellent, very good good; 2 = fair, 3 = poor, very poor). Since our study focuses on the middle-aged and elderly and increasing prevalence of chronic diseases is becoming more and more serious, we also use measures of chronic disease as our third dependent variable. Respondents were considered to have no major chronic disease if they neither reported that a doctor had ever told them they had any of the following chronic diseases: cancer, chronic lung disease, diabetes, heart disease, or stroke, nor obtained a score of four or more on the EURO-D depression scale [33].

#### Cigarette excise tax rate

Cigarette tax rate on the particular cohort was constructed in two steps. The first step was to determine the allocation price according to the retail price of cigarette smoked by respondents. The retail price was based on a CHARLS question “How much does it cost per pack = 20 cigarettes”. The retail price equaled the allocation price plus the allocation-wholesale margin, the wholesale-retail margin, and the value-added tax (VAT = 17%). With cigarette profit margins and VAT deduction, cigarette allocation price could be calculated. Table 1 presents cigarette classification and profit margins. If the retail price was 8 yuan, then the allocate price was 4.50 yuan (8/(1.17*(1 + 10%)*(1 + 38%)))^1^. The next step was to determine the excise tax rate according to the allocation price. If the allocation price was 4.50 yuan, the excise tax rate at the producer level was 30% in 2008. Assuming that the allocation price was 4.50 yuan in 2012, the excise tax rate at the producer level was 36%. Further, a key variable was introduced-the interaction terms between year dummy variables indicating cigarette tax effectiveness and cigarette tax rate on the particular cohort to capture the causal effect of 2009 Chinese cigarette tax. Retail price were also concluded.

#### Individual and state-level characteristics

Demographic variables included age, gender (reference group: female), marital status (single, divorced, widow (common-law marriage is considered as married) was used as the reference) and log of family size which was defined as a census subfamily concludes all related individuals in a household. Educational attainment was defined at three levels: primary school or below, junior high school, and senior high school or above, with primary school or below serving as the reference group. The CHARLS includes extensive income questionnaires to capture extensive sources of household income. The income questions pertain to labor earnings (e.g., wages and salaries or self-employment income), nonlabor income (e.g., interest, dividends, or rental income), private transfer income (e.g., alimony or workers’ compensation; and public transfer income like unemployment, welfare, Social Security). Irregularly received income like stock options and capital gains were not taken into account. Considering the great change in household income concurrent with China’s economic fluctuation, log of household income per capita after the Consumer Price Index revisions, instead of category variables of income was controlled to measure the possible impact of economic development [34]. Another dummy variable, “living in urban area,” indicated whether or not an individual lived in an urban area. Alcohol beverage consumption based on the responses to CHARLS question “Did you drink any alcoholic beverages, such as beer, wine, or liquor, in the last year? How often?” was used to indicate the individual’s current health behaviors. Drinking more than two to three times a week was considered drinking hard liquor.

### Statistical analysis

A multivariable model was constructed as follows: *s*_*ijk*_ = *β*_0_ + *β*_1_ *YEAR* + *β*_2_ *TAX*_*ijk*_ + *β*_3_ *TAX*_*ijk*_ * *YEAR* + *β*_4_ *X*_*ijk*_ + *μ*_*i*_ + *ε*_*ijk*_

Where

*s*_*ijk*_ = smoking status/health for individual i living in province j in year k.

*TAX*_*ijk*_ = smoking tax individual i living in province j in year k pay for,

*YEAR* = the year dummy variable present before or after;

*TAX*_*ijk*_ * *YEAR* = the interaction variable measured the causal effect of smoking tax change in 2009 on smoking;

*X*_*ijk*_ = a vector of observed individual characteristics, such as age, marital status, education, income, et al..

*μ*_*i*_ = individual random effect, and ε = the error term

Since smoking behavior and self reported health outcomes was measured by category outcomes, random effect ordered Logit/Logit model were applied to analyze the relation between cigarette tax adjustment in 2009 and smoking behavior, health outcomes. One of the assumptions underlying ordered logit regression is that the relationship between each pair of outcome groups is the same. The parallel regression assumption was accepted by smoke variable, not by self-reported health. Ordered Logit model were used to estimate smoking status and a generalized ordered logit model was applied to estimate the efforts of cigarette tax adjustment on self-reported health. All analyses were performed using the STATA version 12.0.

## Results

Table 2 shows descriptive statistics for the variables. The average age of current smokers was 61 years. On average, they smoked 12 cigarettes daily; 34% smoked fewer than 10 cigarettes per day (including quitter in 2012), 24% smoked more than 10 but fewer than 20 cigarettes per day, and 42% smoked more than 20 cigarettes per day. 1% smokers regarded their health as excellent; 7% smokers identified their health as very good; 20% smokers identified their health as good; 36% as fair; 30% as poor, 6% as very poor. Smokers smoked fewer cigarettes and rated their health better in 2012 than in 2008, and these differences were statistically significant.

**Table 2:** Individual socioeconomic and life style characteristics

	All sample		2008		2012	
	Mean	Std. Error	Mean	Std. Error	Mean	Std. Error
Daily consumed cigarettes No.	12.59	12.82	13.68***	12.77	11.28	12.76
Self reported health						
Excellent	0.01	0.11	0.02***	0.14	0.01	0.07
Very good	0.07	0.26	0.07***	0.26	0.08	0.27
Good	0.20	0.40	0.18***	0.38	0.22	0.42
Fair	0.36	0.48	0.31***	0.46	0.40	0.49
Poor	0.30	0.46	0.34***	0.47	0.26	0.44
Very poor	0.06	0.23	0.08***	0.27	0.03	0.16
Chronic disease	0.68	0.47	0.63***	0.48	0.73	0.45
Demographics						
Age	60.58	9.56	58.77	9.42	62.63	9.31
Gender	0.96	0.19	0.96	0.19	0.96	0.19
Marital status	0.84	0.36	0.85	0.36	0.84	0.37
Family size	3.09	1.44	3.11	1.47	3.06	1.42
Educational attainment						
Primary school and below	0.71	0.45	0.70	0.46	0.71	0.44
Middle school	0.19	0.39	0.20	0.40	0.19	0.39
High school and above	0.10	0.30	0.10	0.30	0.10	0.30
Household income	33059.64	82253.68	32871.38	80275.68	33083.6	82282.84
Living in urban area	0.25	0.43	0.25	0.43	0.25	0.43
Life style						
Drinking hard liquor	0.50	0.50	0.51	0.50	0.49	0.50

Chi-square test showed that smokers smoked fewer cigarettes and rated their health better in 2013 than in 2008, and these differences were statistically significant. *p < 0.1, **p < 0.05,***p < 0.01

Table 3 shows the multiple regression results of the relationship between cigarette tax adjustment in 2009 and smoking behaviors/health after controlling for socioeconomic status, alcohol consumption, and other demographic characteristics. The negative association of retail price, excise tax rates at the producer level and the number of cigarettes smoked daily/ health supported the hypothesis that taxation is an effective measure for tobacco control. However, it was clearly seen that the number of cigarettes smoked daily tended to increase and health became worse after the adjustment in excise tax rates at the producer level in 2009, which is reflected in the positive and significant coefficients of the interaction terms.

**Table 3:** Effects of the Excise Tax Rate at producer level in 2009 on smoking behaviors and health status

	Daily consumed cigarettes number		Self-reported health				Chronic disease	
			Fair health		Poor health			
	Coef.	Std. Err.	Coef.	Std. Err.	Coef.	Std. Err.	Coef.	Std. Err.
Interaction between tobacco tax and intervention time	5.02***	1.56	2.27	1.69	4.47**	2.18	13.19***	4.36
Tobacco tax	−10.15***	1.47	−0.61	1.55	−1.58***	1.83	−10.90**	4.48
Intervention time	−0.45***	0.14	−0.27	0.16	−0.62***	0.20	−0.98**	0.39
Cigarette price	−0.08***	0.01	−0.05***	0.01	−0.06***	0.02	−0.01	0.05
Age	−2.72***	0.36	1.72***	0.40	2.64***	0.40	13.47***	2.23
Gender	0.62**	0.26	0.04	0.29	0.63**	0.31	−2.16	1.40
Marital status	0.28*	0.15	−0.03	0.18	0.09	0.17	0.66	0.78
Family size	0.12	0.11	0.24*	0.13	−0.06	0.12	1.06*	0.63
Educational attainment								
Middle school	−0.63***	0.17	0.38***	0.19	−0.12	0.20	−2.55***	0.96
High school and above	−0.01	0.18	0.15	0.21	−0.04	0.22	−1.19	0.94
Household income	−0.12***	0.04	−0.08*	0.05	−0.16***	0.05	−0.04	0.25
Living in urban area	0.01	0.11	−0.01	0.12	−0.03	0.13	1.02	0.66
Drinking hard liquor	0.41***	0.1	−0.23**	0.11	−0.19*	0.11	0.05	0.38
Prob > chi2	*p* < 0.01		*p* < 0.01		*p* < 0.01		*p* < 0.01	

*p < 0.1, **p < 0.05,***p < 0.01

“Self-reported health” was recoded into an ordinal variable from 1 to 3 representing the level of health (1 = excellent, very good good; 2 = fair, 3 = poor, very poor)

Table 4 presents the marginal effects of cigarette tax adjustment in 2009 on smoking behavior and health. Cigarette tax adjustment in 2009 resulted in the decrease in the likelihood of smoking 0–10 cigarettes per day by 1.06%; the increase in the likelihood of those smoking 11–20 cigarettes per day by 0.44%; and, those smoking 20 cigarettes or more by 0.63%; the decrease in the likelihood of self-reported good health by 0.47%; fair health by 0.37%; the increase in the likelihood of self-reported poor health by 0.84%; the increase in the prevalence of chronic disease by 1.34%.

**Table 4:** Marginal Effects of the Excise Tax Rate at producer level in 2009 on smoking behaviors and health status

	Daily consumed cigarettes number					
	Smoked cigarettes 0 – 10 per day		11 – 20 cigarettes per day		More than 20 cigarettes per day	
	Marginal effects	Std. Err.	Marginal effects	Std. Err.	Marginal effects	Std. Err.
Interaction between tobacco tax and intervention time	−1.06	0.33	0.44	0.14	0.63	0.19
Tobacco tax	2.15	0.30	−0.89	0.14	−1.26	0.18
Intervention time	0.10	0.03	−0.04	0.01	−0.06	0.02
	Self-reported health					
	Good		Fair		Poor	
	Marginal effects	Std. Err.	Marginal effects	Std. Err.	Marginal effects	Std. Err.
Interaction between tobacco tax and intervention time	−0.47	0.35	−0.37	0.43	0.84	0.41
Tobacco tax	0.12	0.32	0.17	0.36	−0.30	0.34
Intervention time	0.06	0.03	0.06	0.04	−0.12	0.04
		Chronic disease				
	Marginal effects	Std. Err.				
Interaction between tobacco tax and intervention time	1.34	0.44				
Tobacco tax	−1.11	0.46				
Intervention time	−0.10	0.04				

## Discussion

This study provides estimates of the tax adjustments in 2009 and smoking behaviors among the Chinese adult population aged over 45. The cigarette tax adjustment in 2009 was associated with individual worsened smoking behaviors and health outcomes. According to most studies, cigarette taxes reduce the smoking consumption. Smoking decreases followed an increase in cigarette taxes, which were in contrast with our results [4–12].

A possible explanation for the association of cigarette tax adjustment in 2009 with an increase in cigarette smoking was that the data sample only included those who were 45 years old or older. It is widely accepted that the price elasticity of smoking links negatively with age [17]. The decision to smoke can be regarded as divestment in the stock of health capital in return for a short-run increase in welfare [35]. Older populations may not see the time preference of the future to the present as significant as young population do, resulting in insensitive smoking consumption, while younger people may cut down their cigarette consumption for this reason. Also, at relative low-income level, younger people may reduce their consumption due to rising smoking expenditure. Our results were consistent with those findings among old people or young people [13–16]. The findings may be generalized to the entire population with caution.

Another possible explanation is that cigarette tax mechanism resulted in small retail price increase and compensatory behavior of smokers [16]. Cigarette allocation prices were increased by 2.6% on average after the tax adjustment of 2009 in China, while the STMA decreased the allocation-wholesale margin. Therefore, the change in retail prices was quite limited [18]. In essence, it could be claimed that the new policy was a profit tax adjustment rather than an excise tax adjustment. In addition, although the average price went up, the retail price for the cigarettes with 5 and 7 yuan allocation price went down, which provide an opportunity for smokers to purchase from low-taxed sources of cigarette. The compensatory behavior of smokers mitigated the impact of any tax induced price increase on smoking and public health outcomes.

Furthermore, considering the tax adjustment is a profit policy, smokers may attribute smoking behavior to support the work of countries in increasing tax revenue, which make smokers be easy to accept behavioral consequences resulting from smoking [18]. Especially in the period, affected by the global economic fluctuations, China experienced economic fluctuations, which may expand the motive to respond to the profit tax adjustment [36]. In addition, smokers may experience short-term benefits from smoking, such as relieves in anxiety, which may help people to ease stresses resulting from a rapid changing environment, which result in increasing smoking [37]. However, mounting evidence has shown substantial costs being associated with smoking in the long-run. Smokers have higher risks of developing debilitating and often serious illnesses such as chronic diseases. The chemicals in tobacco damage the way body heals itself [36, 38]. In short, smoking decreases the individuals’ health capital in the long-run.

Interestingly, although the cigarette tax adjustment in 2009 did not play a positive role in tobacco control, the result suggests that increased tax rate could lead to reduced smoking consumption. The result implies that taxation could work as an effective mean to control tobacco use. However, major reforms to the tax system need to be carried out before any further improvement is expected. To be exact, tax collection at the retail level should be put out of the control of the STMA. By doing this, cigarette tax could be allowed to pass on to overall and each retail price, and then taxation could be an impactful tobacco control tool in China.

This study contributes to the existing literature in three ways. First, at present China does not have a valid assessment tool for the impact of cigarette tax on cigarette smoking. For the first time, the effects of cigarette tax adjustment in 2009 on smoking behaviors and health of a large representative sample were estimated in China. Second, it is well known that smoking is correlated with health outcomes [39]. Only a few studies evaluated the effects of cigarette taxation on health outcomes, and the results were mixed [3]. Sen et al. demonstrated that an increase in cigarette taxes is significantly associated with an increase in the percentage of obese population, while Fishman et al. found that increased cigarette tax decreased smoking-attributable mortality significantly, with large gains in cumulative life-years and quality-adjusted life-years [7, 9, 40]. This is the first study to quantify the effects of cigarette tax adjustment in 2009 on health in China. Third, the previous reports constructed cigarette taxes at the state level. However, in the present study, with the retail price, the cigarette tax rate was constructed at the individual level, allowing for an accurate estimation of the tax effect.

Our study has limitations. A limitation of this study is due to the small population. The final sample size is only 1366 respondents, which is not national-represented. Although the two provinces were chosen to get at extremes within China, the finding should be applied to all the country with caution. Smoking population in China is known to be high in men but low in women, the effectiveness of tax-adjustment on cigarettes could be influenced on gender-difference [38]. Concurrent with the small population with 4% of female smokers, sex-specific analysis was incapable to be performed.

Another limitation of this study was that the data could not account for the instant effects of the tax adjustment in 2009 on smoking behavior and health outcome, since the survey was not conducted in from 2010–2011. On average, the smokers in the sample selected had been smoking cigarettes for more than 20 years. Tobacco contains nicotine, which is a highly addictive substance. Hence, it may take a long time for the smokers to quit smoking or decrease the number of cigarettes smoked daily in response to the cigarette tax adjustment in 2009. Also, the effectiveness of tax for cigarettes could not evaluated only by the impact on current smoker. Kostova et al. found that initiation rates fall in response to higher prices [41]. However, among the respondents aged over 45 years old in our sample, the initial rate is quite low. Between year 2008–2012, there are only 13 respondents start to smoke.

## Conclusion

This is the first study to estimate the effects of cigarette tax adjustment in 2009 in China. The smoke tax adjustment in 2009 increased the number of cigarettes smoked daily, and worsened individual health outcomes. The proposed cigarette tax levied at the retail level can reduce the STMA’s control overall and each price and increase the influence of the market on cigarette consumption in China.

## Abbreviations

CHARLS: China Health and Retirement Longitudinal Study
CNTC: Chinese National Tobacco Company
FCTC: Framework Convention on Tobacco Control
PPS: Probability-proportionate-to-size
STMA: State Tobacco Monopoly Administration
VAT: Value-added tax
WHO: World Health Organization

## Footnotes

1 If the allocation-wholesale margin was 28%, the allocate price was 4.86, above 3 yuan; if the allocation-wholesale margin was 43%, the allocate price was 4.35, below 5 yuan. So the allocation-wholesale margin must be 38%, and the allocate price was 4.50 yuan.
